# Association between residential greenness and anemia risk among women of reproductive age in low- and middle-income countries

**DOI:** 10.3389/fpubh.2026.1828024

**Published:** 2026-07-06

**Authors:** Yanwen Cao, Bangjie Guo, Zunyan Chu, Yingying Cao, Xinyi Li, Qi Zhao, Xiaoying Lin, Tao Huang

**Affiliations:** 1 Department of Epidemiology, School of Public Health, Cheeloo College of Medicine, Shandong University, Jinan, Shandong, China; 2 Shandong University Climate Change and Health Centre, Shandong University, Jinan, Shandong, China; 3 Faculty of Health, Deakin University, Melbourne, VIC, Australia; 4 The Second Hospital, Cheeloo College of Medicine, Shandong University, Jinan, Shandong, China; 5 Medical Integration and Practice Center, Cheeloo College of Medicine, Shandong University, Jinan, Shandong, China

**Keywords:** greenness, anemia, women of reproductive age, low- and middle-income countries, NDVI, EVI

## Abstract

**Background:**

Anemia is a major public health burden in low- and middle-income countries (LMICs), particularly among women of reproductive age (WRA). While biological causes are established, evidence on environmental factors like residential greenness is limited.

**Methods:**

We analyzed data from 1,074,790 WRA across 40 LMICs using Demographic and Health Surveys (2005–2019), linked with Normalized Difference Vegetation Index (NDVI) and Enhanced Vegetation Index (EVI) across multiple buffer sizes (500–3,000 m). Associations between greenness and anemia were assessed using a generalized linear mixed-effects model with adjustment for demographic, socioeconomic, and environmental confounders. Under a hypothetical scenario, we further estimated the fraction and number of anemia cases associated with greenness exposure.

**Results:**

High greenness was consistently associated with lower anemia risk across all buffer sizes of NDVI and EVI. Linear models suggested that the odds ratio (OR) of anemia was 0.932 [95% confidence interval (95% CI): 0.929–0.936] for each 0.1-unit increase in NDVI-1000 m. Non-linear models further indicated that the inverse association became more pronounced at higher greenness levels. Stratified analyses showed that the effect size of greenness varied across subgroups defined by BMI, breastfeeding status, wealth index, residence, and education. Under the hypothetical greenness improvement scenario, 2.25% (95% CI: 1.20–3.30%) of anemia cases among WRA were associated with greenness levels relative to the target NDVI-1000 m level, with substantial variation across countries and exposure buffers.

**Conclusion:**

Higher residential greenness was associated with lower anemia risk among WRA in LMICs. These findings suggest that residential greenness may represent a potentially relevant environmental factor in future research and public health strategies addressing anemia-related environmental determinants.

## 1 Introduction

Anemia contributes substantially to global disease burden, with women of reproductive age (WRA, 15–49 years) particularly susceptible due to physiological status such as menstruation, pregnancy, and childbirth (1, 2). In 2019, over 30% of WRA worldwide were affected by anemia, and the burden exhibited large geographic variation (3). For example, cases of WRA with anemia during 2000–2019 remained 36.1–39.0 million in high-income countries, which increased significantly from 246.6 to 326.1 million in low- and middle-income countries (LMICs) (4). Anemia can cause fatigue and cognitive impairment, and is strongly associated with adverse pregnancy outcomes (e.g., preterm birth, low birth weight, and higher maternal and infant mortality) (2). Given the heavy health and socioeconomic burden of anemia in LMICs, there is a critical need to clarify its modifiable risk factors.

Nutritional deficiencies, infections, inflammation, and genetic hemoglobin disorders are well recognized contributors to anemia in LMICs (5). In comparison, environmental and social determinants have received less attention. Among modifiable environmental exposures, residential greenness attracts growing interest from perspectives of epidemiology and urban planning because of its potential health benefits (6, 7). Numerous epidemiological studies have applied satellite derived vegetation indices, particularly the normalized difference vegetation index (NDVI) and enhanced vegetation index (EVI), to quantify the impact of residential greenness exposure (8–10). For example, a nationwide cohort study in China found that residing in the highest quartile of NDVI within a 1,250 m buffer was associated with approximately 30% lower all-cause mortality among oldest-old adults than those in the lowest quartile (7). Higher residential greenness, measured using NDVI and EVI, has also been associated with favorable cardiovascular, metabolic, mental, and reproductive health outcomes across different populations (6, 7, 11).

The potential health effects of residential greenness may operate through several pathways, including reduced physiological stress, systemic inflammation, and heat exposure, alongside increased physical activity and improved air quality (7, 11, 12). Some of these pathways are directly implicated in anemia development (13). Despite this biological plausibility, whether residential greenness is associated with anemia risk remains largely unexplored. Moreover, previous evidence suggests that the health effects of greenness may vary by individual and household characteristics (e.g., age, education, and socioeconomic status) (14). However, existing studies are limited by small sample sizes, single-country settings, and insufficient consideration of population heterogeneity. Large-scale, standardized, population-based data with broad geographic coverage are therefore needed for this research gap.

The Demographic and Health Surveys (DHS) provide harmonized household, sociodemographic, and health data across many LMICs, using survey designs that are nationally representative within participating countries. Their cluster-based geolocation further allows integration with satellite-derived exposure metrics, making DHS an important platform for examining environmental determinants of health in resource-limited settings (15). Previous DHS-based studies have demonstrated the feasibility of incorporating environmental exposures into analyses of physiological and developmental outcomes. For example, fine particulate matter exposure has been associated with increased anemia among WRA, whereas climate-related environmental stressors, such as compound drought and heatwave events, have been linked to adverse child health outcomes in LMICs (16, 17). Using DHS data, this study assessed the association between residential greenness and anemia risk of WRA from 40 LMICs between 2005 and 2019. We further estimated the attributable fraction (AF) and attributable number (AN) of anemia cases under a hypothetical greenness improvement scenario using a counterfactual framework. This study aims to improve understanding of residential greenness as a potential environmental determinant of anemia in LMICs.

## 2 Materials and methods

### 2.1 Study population and DHS data source

This cross-sectional study used representative data from DHS, which are conducted approximately every five years in over 90 LMICs (15). The DHS uses a stratified two-stage cluster sampling design and collects information on household demographics, socioeconomic status, reproductive history, nutrition, and health through standardized questionnaires and biomarker assessments. Since 2005, most DHS surveys recorded the geographic coordinates of household clusters using global positioning system (GPS) devices, enabling linkage with environmental exposure data. Further details about DHS surveys are available on the official website^1^.

The study population was WRA (15–49 years) residing in these LMICs. Participants were excluded if without valid hemoglobin measurements, GPS data, environmental exposure data, or complete information on key covariates. The list of included countries is shown in Supplementary (Text S1), and the exclusion flowchart of population is shown in Supplementary Figure S1. All procedures and questionnaires for standard DHS surveys have been reviewed and approved by ICF Institutional Review Board, and the respondents have been aware of the informed consent statement before each interview or biomarker test.

### 2.2 Assessment of residential greenness exposure

NDVI and the EVI were applied to measure greenness exposure. NDVI is one of the most widely used vegetation indices, which can quickly show where vegetation is located, but it is sensitive to clouds and aerosols (15). EVI was later developed to reduce the effects of background noise, air conditions, and soil (6). Both data were obtained from the Moderate Resolution Imaging Spectroradiometer (MODIS) at a spatial resolution of 250 × 250 m.

NDVI and EVI values range from −1 to 1. Values below 0 generally indicate non-vegetated surfaces, while positive values reflect vegetation presence, with higher values representing greater vegetation density (6, 18). In this study, NDVI and EVI values less than 0 were set to 0 to ensure that exposure metrics represented actual vegetation. To capture greenness at both neighborhood and larger spatial scales, we calculated annual average NDVI and EVI within buffer sizes of 500 m, 1,000 m, 2000 m, and 3,000 m around each participant’s residence (hereafter referred to as NDVI_-500m_, NDVI_-1000m_, NDVI_-2000m,_ and NDVI_-3000m_, with the same naming convention applied to EVI). These buffer sizes roughly correspond to walking times of 10, 15, 20, and 30 min, respectively (19, 20). Multi-buffer analyses were conducted to assess the robustness of our findings.

### 2.3 Assessment and definition of anemia

The hemoglobin concentrations (g/dL) of WRA were measured through finger prick using the HemoCue blood hemoglobin testing system. Hemoglobin counts were adjusted for altitude [see details in Supplementary (Text S2)]. According to World Health Organization (WHO) criteria, anemia was defined in pregnant women as hemoglobin concentration <11.0 g/dL and in non-pregnant women as <12.0 g/dL at sea level (21). Blood testing was voluntary, and respondents received their results of anemia test immediately after assessment.

### 2.4 Assessment of potential confounding factors

Following previous studies (22, 23), a set of covariates were included to reduce potential confounding. Individual-level covariates included age, body mass index (BMI), education level (no education, primary, secondary, or higher), marital status (never, current, or former), and breastfeeding status (yes or no). Specifically, BMI was categorized as underweight (<18.5 kg/m^2^), normal (18.5–24.9 kg/m^2^), overweight (25.0–29.9 kg/m^2^), or obese (≥30 kg/m^2^). Household-level covariates included place of residence (urban or rural), wealth index (poorest, poorer, middle, richer, richest), family size, age and gender of the household head, and whether the household had health insurance. Environmental and household-infrastructure covariates included type of cooking energy (clean or unclean), source of drinking water (clean or unclean), availability of a flushable toilet and annual average PM_2.5_ concentration. PM_2.5_ data were obtained from the Atmospheric Composition Analysis Group at a 1 × 1 km spatial resolution and then matched to each participant’s residential address. Other covariates were obtained from the DHS questionnaire.

### 2.5 Statistical analysis of greenness–anemia associations

Continuous variables were summarized as means with standard deviations (SD). Categorical variables were presented as frequencies and percentages. Inter-group differences in population characteristics were assessed using chi-square test for categorical variables and Student’s t-test for continuous variables.

Generalized linear mixed-effects models (GLMMs) were used to estimate the associations between residential greenness and anemia, with country included as a random-effect variable to account for clustering. Model 1 was an unadjusted model. Model 2 adjusted for a subset of covariates (e.g., age, residence, and wealth index). Model 3 was fully adjusted for all individual-, household-, and environmental-level covariates described in Section 2.4. The 1,000 m buffer was selected as the primary exposure scale, which represents neighborhood-level greenness. This buffer is broadly consistent with the walking range defined in the “15-min city” framework, and has been commonly used in previous epidemiological studies (6, 20, 24, 25).

To explore potential non-linear relationship between greenness and anemia, linear and non-linear terms of NDVI or EVI were applied for each model, respectively. For linear models, results were reported as odds ratios (ORs) and 95% confidence intervals (CIs) of anemia in relation to a 0.1-unit increase in NDVI or EVI. Greenness exposures were also categorized into tertiles, with the lowest tertile (Q1) used as the reference group. The non-linear term was fitted using a restricted cubic spline (RCS) function with three knots placed at the 10th, 50th, and 90th percentiles based on previous literature (6, 26). The likelihood ratio test was used to compare linear and non-linear models.

To assess potential effect modification, stratified analyses were performed by age, BMI, pregnancy status, breastfeeding status, wealth index, place of residence, and education level. All stratified models were adjusted for the same covariates as the fully adjusted model (i.e., model 3). We employed a formula [ $(E1-E2)\pm \sqrt{(\mathrm{SE}1)2+(\mathrm{SE}2)2}$ ], utilizing point estimate (E) and standard error (SE), to assess the statistical significance of differences across subgroups (27).

### 2.6 Hypothetical anemia burden estimation under greenness improvement

To explore the potential public health relevance, we conducted a hypothetical analysis to estimate the AF and AN of anemia cases under improved greenness exposure (6, 28). The burden estimation was limited to 2015 due to the availability of high-resolution population estimates of WRA from WorldPop (29). We used 2015 as a descriptive reference year to approximate population distribution and environmental conditions during the study period. Based on the exposure–response function (ERF) extracted from the non-linear model, we estimated the number of anemia cases among WRA hypothetically attributable to greenness levels below the target. Specifically, we defined a hypothetical improvement scenario in which residential greenness was increased to a target level, defined as the mean NDVI or EVI across all spatial cells.

To account for the spatial heterogeneity of exposure and the distribution of WRA, NDVI/EVI rasters at 250 × 250 m resolution were resampled to 1 × 1 km resolution to match the population grids from WorldPop. Country-level anemia prevalence was obtained from the WHO database. We then calculated the AN and AF of anemia using the following steps and equations (Equations 1–4):


$${\mathrm{RR}}_{s,c}=\frac{{\text{OR}}_{s,c}}{(1-P_{c})+P_{c}\times {\text{OR}}_{s,c}}$$ (1)

$${\text{AN}}_{s,c}=W_{s,c}\times P_{c}\times {\mathrm{AF}}_{s,c}=W_{s,c}\times P_{c}\times \frac{{\mathrm{RR}}_{s,c}-1}{{\mathrm{RR}}_{s,c}}\;\;$$ (2)

$$\begin{array}{c} {\text{AN}}_{c}=\sum_{S}{\text{AN}}_{s,c} \end{array}$$ (3)

$$\begin{array}{c} {\mathrm{AF}}_{c}=\frac{{\text{AN}}_{c}}{N_{c}}=\frac{{\text{AN}}_{c}}{\sum_{s}(W_{s,c}\times P_{c})} \end{array}$$ (4)

In these equations, s and c represent pixels and countries, respectively. ${\text{OR}}_{s,c}$ is the OR of anemia estimated from the ERF for a given greenness level and buffer size in each pixel. If the greenness level of a pixel exceeds the target (defined as the mean NDVI or EVI across all pixels), ${\text{OR}}_{s,c}$ was set to 1, assuming no additional risk. ${\mathrm{RR}}_{s,c}$ is the relative risk (RR) converted from ${\text{OR}}_{s,c}$ using a standard formula. $P_{c}$ is the national anemia prevalence in 2015, and $W_{s,c}$ is the pixel-specific number of WRA based on WorldPop estimates. ${\text{AN}}_{s,c}$ and ${\mathrm{AF}}_{s,c}$ represent the number and fraction of anemia cases that could potentially be reduced via improving greenness exposure to the target level within each pixel. Finally, we calculated the total modifiable burden across all countries using Equations 5 and 6:


$$\begin{array}{c} {\text{AN}}_{\text{total}}=\sum_{c}\;{\text{AN}}_{c} \end{array}$$ (5)

$$\begin{array}{c} {\mathrm{AF}}_{\text{total}}=\frac{{\text{AN}}_{\text{total}}}{\sum_{c}\;\sum_{s}(W_{s,c}\times P_{c})} \end{array}$$ (6)

All analyses were performed using R version 4.3.1 software, with two-sided tests for all statistical evaluations. *p* values <0.05 were considered statistically significant.

## 3 Results

### 3.1 Summary characteristics

This study included 1,074,790 WRA (mean age: 29.65 ± 9.79 years) from 40 LMICs (Supplementary Figure S1; Table 1), with 46.74% diagnosed with anemia. Among all participants, 5.48% were pregnant, 18.47% were currently breastfeeding, 17.12% were underweight and 26.72% had no formal education. Over half lived in rural areas (66.73%) and 19.63% belonged to the poorest household wealth group. Mean NDVI and EVI values ranged between 0.461–0.468 and between 0.287–0.291 across buffers, respectively (Supplementary Table S1). The two indices showed similar geographic patterns, with higher greenness levels observed in central and west Africa, parts of south Asia, and the Amazon basin (Supplementary Figure S2).

**Table 1:** General characteristics of study participants.

Characteristic	All participants	Incident anemia cases	Non-cases	*P* for differences
Number of participants, mean (SD)	1,074,790 (100.00)	502,312 (46.74)	572,478 (53.26)	
Individual-level variables				<0.001
Age (year), mean (SD)	29.65 (9.79)	29.54 (9.73)	29.75 (9.85)	
BMI (kg/m^2^), *n* (%)				<0.001
Underweight, *n* (%)	184,018 (17.12)	101,010 (20.11)	83,008 (14.50)	
Normal	641,685 (59.70)	304,779 (60.68)	336,906 (58.85)	
Overweight	177,546 (16.52)	70,660 (14.07)	106,886 (18.67)	
Obese	71,541 (6.66)	25,863 (5.15)	45,678 (7.98)	
Highest education level, *n* (%)				<0.001
No education	287,202 (26.72)	152,229 (30.31)	134,973 (23.58)	
Primary	223,692 (20.81)	93,501 (18.61)	130,191 (22.74)	
Secondary	459,310 (42.73)	213,139 (42.43)	246,171 (43.00)	
Higher	104,586 (9.73)	43,443 (8.65)	61,143 (10.68)	
Marital status, *n* (%)				<0.001
Never	279,236 (25.98)	121,759 (24.24)	157,477 (27.51)	
Current	731,474 (68.06)	352,553 (70.19)	378,921 (66.19)	
Former	64,080 (5.96)	28,000 (5.57)	36,080 (6.30)	
Breastfeeding, *n* (%)				<0.001
Yes	198,472 (18.47)	97,176 (19.35)	101,296 (17.69)	
No	876,318 (81.53)	405,136 (80.65)	471,182 (82.31)	
Pregnant, *n* (%)				<0.001
Yes	58,920 (5.48)	42,066 (8.37)	16,854 (2.94)	
No	1,015,870 (94.52)	460,246 (91.63)	555,624 (97.06)	
Residence, *n* (%)				<0.001
Rural	717,188 (66.73)	347,708 (69.22)	369,480 (64.54)	
Urban	357,602 (33.27)	154,604 (30.78)	202,998 (35.46)	
Wealth index, *n* (%)				<0.001
Poorest	210,992 (19.63)	109,559 (21.81)	101,433 (17.72)	
Poorer	220,465 (20.51)	106,419 (21.19)	114,046 (19.92)	
Middle	218,832 (20.36)	101,712 (20.25)	117,120 (20.46)	
Richer	213,000 (19.82)	95,364 (18.99)	117,636 (20.55)	
Richest	211,501 (19.68)	89,258 (17.77)	122,243 (21.35)	
Family size				<0.001
Age of household head (year), mean (SD)	46.48 (13.40)	46.40 (13.32)	46.55 (13.47)	
Gender of household head, *n* (%)				<0.001
Male	868,126 (80.77)	411,865 (81.99)	456,261 (79.70)	
Female	206,664 (19.23)	90,447 (18.01)	116,217 (20.30)	
Health insurance covered, *n* (%)				<0.001
No	180,832 (16.82)	82,443 (16.41)	98,389 (17.19)	
Yes	893,958 (83.18)	419,869 (83.59)	474,089 (82.81)	
Type of cooking energy, *n* (%)				<0.001
Unclean	339,992 (31.63)	150,020 (29.87)	189,972 (33.18)	
Clean	734,798 (68.37)	352,292 (70.13)	382,506 (66.82)	
Type of drinking water, *n* (%)				<0.001
Unclean	864,035 (80.39)	408,997 (81.42)	455,038 (79.49)	
Clean	210,755 (19.61)	93,315 (18.58)	117,440 (20.51)	
Flushable toilet used, *n* (%)				<0.001
No	450,345 (41.90)	200,320 (39.88)	250,025 (43.67)	
Yes	624,445 (58.10)	301,992 (60.12)	322,453 (56.33)	

SD, Standard deviation; BMI, Body mass index.

### 3.2 Associations between greenness exposure and anemia risk

Both linear and non-linear models showed that higher values of NDVI and EVI were significantly associated with lower anemia across all buffer sizes from 500 m to 3,000 m (Table 2; Supplementary Figure S3). In the fully adjusted model, each 0.1-unit increment in NDVI_-1000m_ was associated with a 6.8% lower risk of anemia (OR = 0.932, 95% CI: 0.929–0.936). Similar associations were observed for EVI_-1000m_. The association became stronger at larger buffer sizes, such that the OR decreased from 0.938 (95% CI: 0.935–0.942) for the 500 m buffer to 0.919 (95% CI: 0.915–0.922) for the 3,000 m buffer. These patterns were consistent in the unadjusted and partially adjusted models, indicating the robustness of main results (Supplementary Table S2). Supplementary Figure S3 illustrates the non-linear ERFs for NDVI and EVI across the four buffer sizes (*p* < 0.001). For instance, anemia risk declined modestly at lower greenness levels, below approximately 0.3 for NDVI_-1000m_ and below approximately 0.2 for EVI_-1000m_, followed by a more pronounced downward trend at higher greenness levels.

**Table 2:** Associations between greenness exposure at different buffer sizes and anemia risk.

Exposure	NDVI		EVI	
	OR (95% CI)	*P*	OR (95% CI)	*P*
500 m buffer				
Tertile 1	Ref. (1)	—	Ref. (1)	—
Tertile 2	0.985 (0.974, 0.995)	0.004	0.982 (0.971, 0.992)	<0.001
Tertile 3	0.874 (0.864, 0.884)	<0.001	0.891 (0.881, 0.901)	<0.001
Per 0.1-unit increment	0.938 (0.935, 0.942)	<0.001	0.923 (0.918, 0.928)	<0.001
1,000 m buffer				
Tertile 1	Ref. (1)	—	Ref. (1)	—
Tertile 2	0.985 (0.975, 0.996)	0.005	0.963 (0.953, 0.973)	<0.001
Tertile 3	0.869 (0.859, 0.879)	<0.001	0.877 (0.868, 0.887)	<0.001
Per 0.1-unit increment	0.932 (0.929, 0.936)	<0.001	0.913 (0.908, 0.919)	<0.001
2,000 m buffer				
Tertile 1	Ref. (1)	—	Ref. (1)	—
Tertile 2	0.985 (0.974, 0.995)	0.003	0.969 (0.959, 0.98)	<0.001
Tertile 3	0.860 (0.851, 0.87)	<0.001	0.873 (0.863, 0.883)	<0.001
Per 0.1-unit increment	0.924 (0.921, 0.928)	<0.001	0.901 (0.896, 0.907)	<0.001
3,000 m buffer				
Tertile 1	Ref. (1)	**—**	Ref. (1)	—
Tertile 2	0.985 (0.975, 0.995)	0.003	0.968 (0.958, 0.978)	<0.001
Tertile 3	0.845 (0.836, 0.855)	<0.001	0.865 (0.855, 0.875)	<0.001
Per 0.1-unit increment	0.919 (0.915, 0.922)	<0.001	0.893 (0.888, 0.898)	<0.001

These estimates are derived from the fully adjusted model. NDVI, normalized difference vegetation index; EVI, enhanced vegetation index; OR, Odds ratio; CI, Confidence interval.

**Figure 1:**
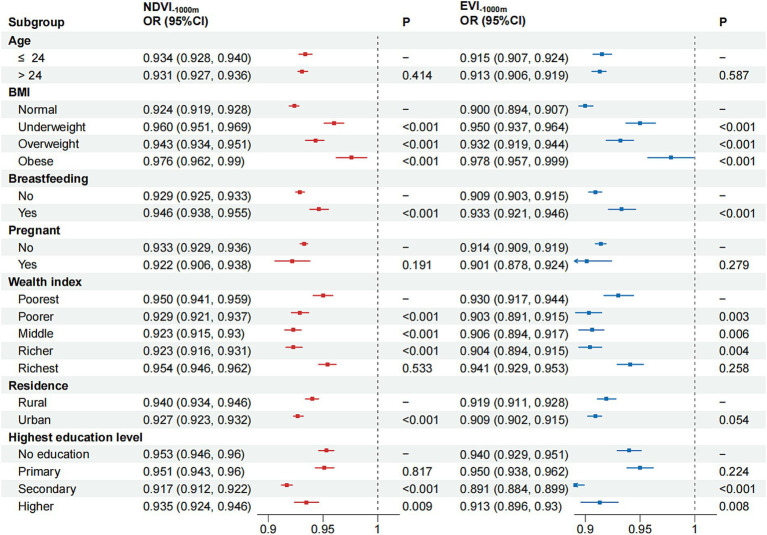
Stratified analysis of the association between greenness (NDVI-1000 m and EVI-1000 m) with anemia risk. NDVI normalized difference vegetation index, EVI enhanced vegetation index.

Subgroup analyses suggested that the strength of association between greenness exposure and anemia risk varied by population characteristics (Figure 1). Briefly, for per 0.1-unit increment in NDVI_-1000m_, the lowest ORs were observed among WRA with normal BMI, those who were not breastfeeding, those with middle-to-richer wealth index, urban residents, and those with secondary or higher education, compared to their respective counterparts. These patterns were generally consistent for both NDVI and EVI across all buffer sizes (Supplementary Figures S4–S6).

### 3.3 Hypothetical anemia burden under improved greenness scenarios

Under the hypothetical greenness improvement scenario, 2.25% (95% CI: 1.20–3.30%) and 1.99% (95% CI: 0.81–3.16%) of anemia cases were associated with low greenness exposure based on NDVI_−1,000_ and EVI_−1,000_, respectively (Supplementary Figure S7). These AFs corresponded to 2.08 million (95% CI: 1.10–3.04 million) and 1.79 million (95% CI: 0.73–2.83 million) cases, respectively. ANs and AFs varied by buffer size. For example, the AF rose from 1.61% (95% CI: 0.48–2.75%) for EVI_-500m_ to 2.55% (95% CI: 1.30–3.79%) for EVI_-3000m_. Corresponding ANs increased from 1.44 million (95% CI: 0.43–2.47 million) to over 2.29 million (95% CI: 1.17–3.40 million).

**Figure 2:**
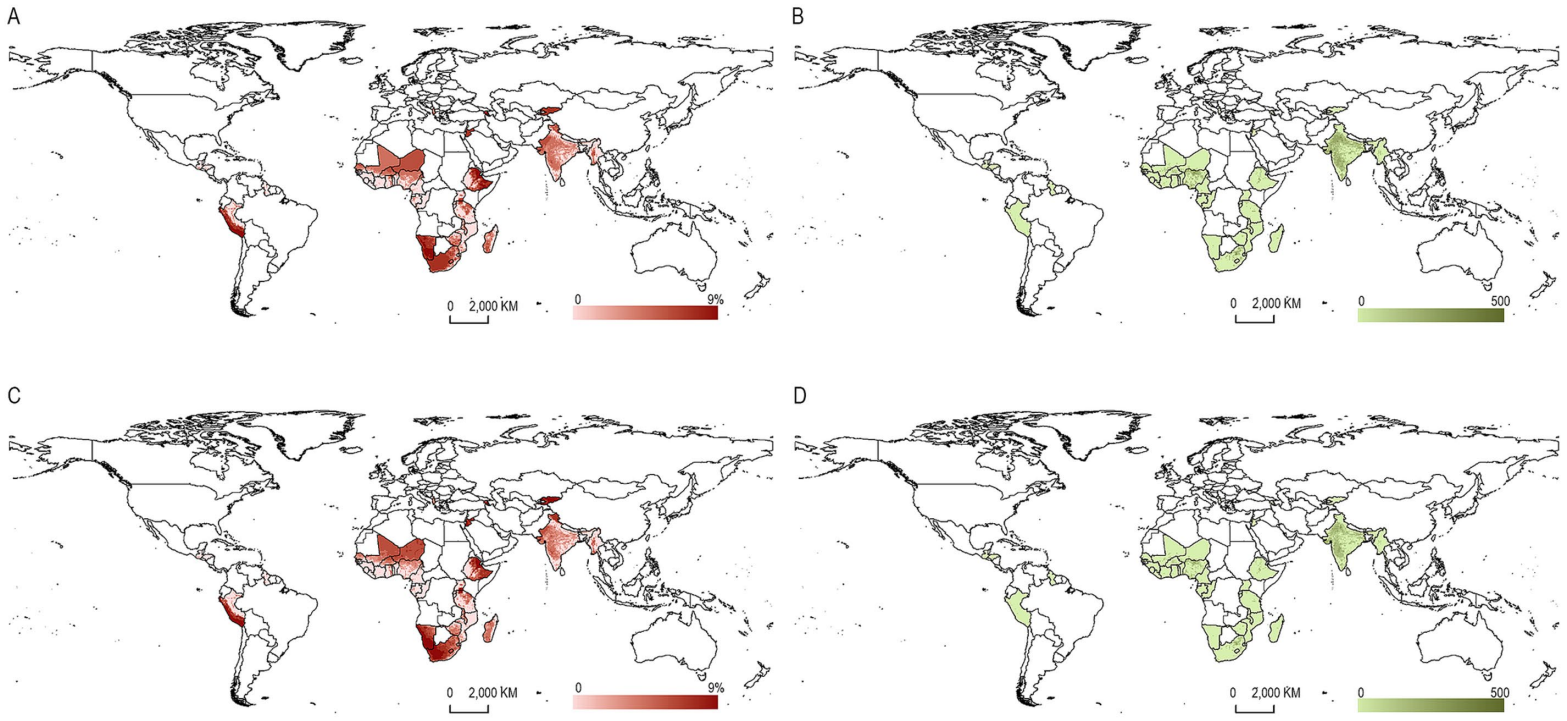
Distribution of attributable fraction (AF) and number (AN) by achieving greenness targets of anemia in 2015. **(A)** AF of NDVI-1000 m, **(B)** AN of NDVI-1000 m, **(C)** AF of EVI-1000 m, **(D)** AN of EVI-1000 m. NDVI, normalized difference vegetation index; EVI, enhanced vegetation index.

**Figure 3:**
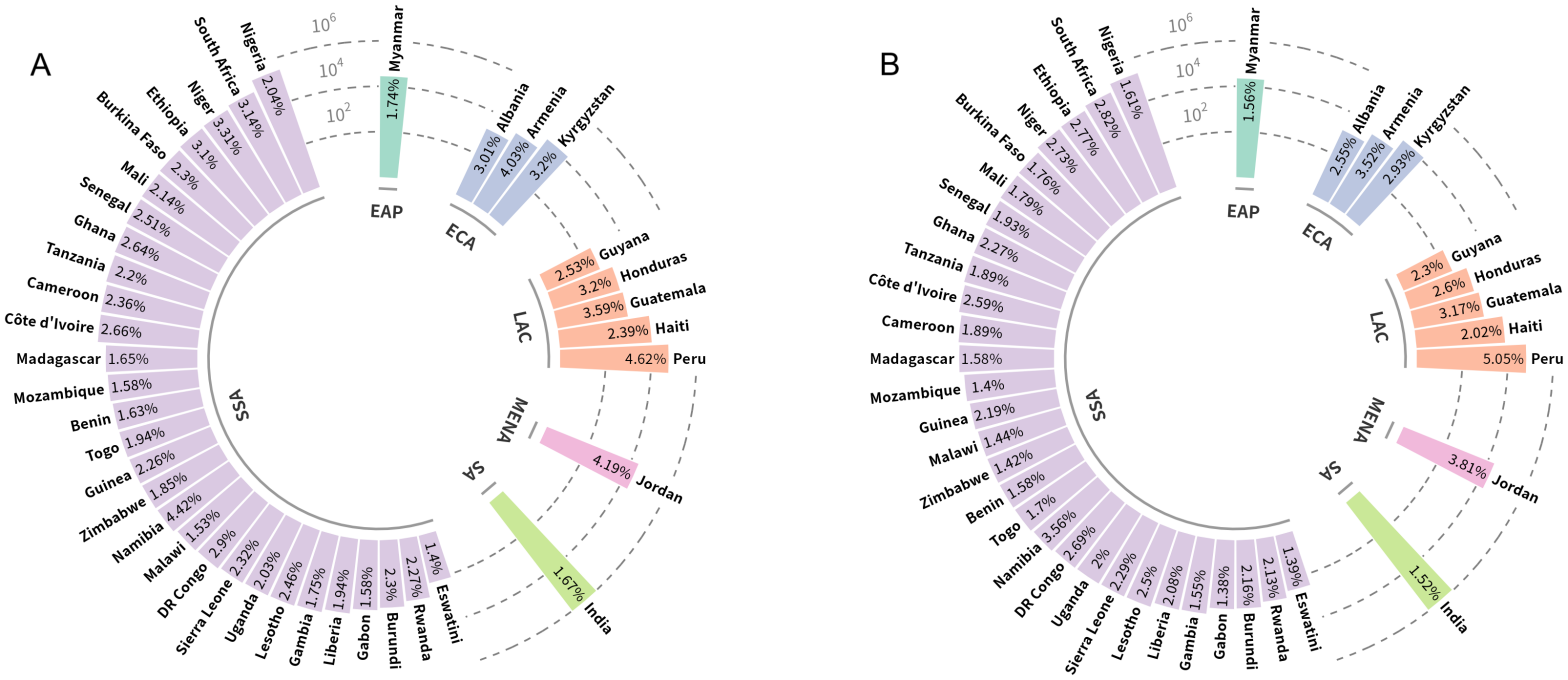
Attributable fraction (AF, %) and number (AN, log scale) by achieving greenness targets of anemia across 40 countries in 2015. **(A)** AF and AN of NDVI-1000 m, **(B)** AF and AN of EVI-1000 m. Bar height represents AN, while the number labeled inside each bar indicates AF. NDVI, normalized difference vegetation index; EVI, enhanced vegetation index; EAP, East Asia and Pacific; ECA, Europe and Central Asia; LAC, Latin America and the Caribbean; MENA, Middle East and North Africa; SA, South Asia; SSA, Sub-Saharan Africa.

Regionally, the highest AFs were observed in parts of southern and central Africa, and the northwest of south America (Figures 2, 3; Supplementary Table S3). The spatial distribution of ANs did not entirely overlap with the AF hotspots. For example, Armenia had an AF of 4.03% (95% CI: 2.48–5.56%) under the NDVI_-1000m_ model, but the corresponding AN was relatively low. By contrast, India had a lower AF of 1.67% (95% CI: 0.76–2.57%) but accounted for over 1 million AN. Overall, South Asia and Sub-Saharan Africa contributed the largest ANs, while Latin America and the Caribbean, Middle East and North Africa, and Europe and Central Asia had relatively higher AFs. EVI-based estimates showed similar spatial patterns.

## 4 Discussion

### 4.1 Main findings

This study identified a significant negative correlation between residential greenness and anemia risk among WRA in 40 LMICs. The association was consistent across different greenness metrics (NDVI and EVI) under various buffer sizes (500 m–3,000 m). Effect heterogeneity of greenness was observed across subgroups with varied socioeconomic and physiological status. Under the hypothetical analysis, low greenness exposure was associated with a potentially substantial anemia burden, with marked variation across countries. These findings provide novel multi-country evidence supporting the role of greenness improvement in addressing nutritional disorders.

### 4.2 Interpretation of findings and potential mechanisms

To date, direct epidemiological evidence on the association between residential greenness and anemia risk remains limited, with most available studies conducted in single-country settings. In India, a recent nationwide analysis based on the fourth and fifth rounds of National Family Health Surveys reported that each standard deviation increase in EVI was associated with slightly lower OR of anemia among WRA [0.992 (95% CI: 0.986–0.998)] (8). Evidence from intervention studies is also limited and not entirely consistent. For example, a study in Nepal reported that school and household garden programs were associated with improved hemoglobin levels among children below 12 years, potentially through better food and hygiene practices, but no significant reduction in anemia prevalence was observed (30). These inconsistent findings may reflect variation in study populations, greenness metrics, exposure settings, and environmental and healthcare conditions. Compared with these localized studies, our multi-country analysis across 40 LMICs provides broader evidence that higher residential greenness is associated with lower anemia risk among WRA across diverse socioeconomic and environmental settings.

Our findings are consistent with broad literature indicating that exposure to greenness may benefit a range of health outcomes through overlapping biological pathways. For example, several epidemiological studies have indicated that high residential greenness is associated with reduced risks of cardiovascular diseases, metabolic disorders, and adverse pregnancy outcomes (7, 11, 12, 31). These conditions often share common biological pathways with anemia, such as systemic inflammation, oxidative stress, and impaired oxygen transport, supporting the plausibility of our findings. In addition, indirect evidence from studies focusing on other environmental exposures further aligns with our results (22, 24, 32). For instance, exposure to PM_2.5_ has been associated with elevated anemia risk through inflammatory and oxidative mechanisms (22, 32). Similarly, extreme heat exposure has been linked to hemoconcentration, increased blood viscosity, and impaired erythropoiesis (24), thereby contributing to anemia development. Residential greenness is known to mitigate the effects of air pollution and heat exposure, and promote physical activity, all of which are protective against pathways leading to anemia (7, 11, 12). Therefore, although few studies have directly assessed the relationship between greenness and anemia, the existing literatures provide substantial supportive evidence for the observed associations in our study.

### 4.3 Effect patterns and population heterogeneity

Our ERF analysis revealed a non-linear association between residential greenness and anemia risk. The association exhibited only marginal risk reduction at lower greenness levels, with more pronounced declines emerging beyond moderate exposure (18, 33). This pattern suggests a potential threshold effect: Insufficient greenness exposure may fail to provide adequate mitigation of physiological stress or facilitate health-promoting behaviors essential for the prevention and control of anemia. Similar results have been observed previously. Analysis of UK Biobank data showed sustained mental health benefits from residential greenness, where significant reductions emerged only at higher exposure levels (18). A nationwide cohort study of women in the United States reported an inverse association between residential greenness, measured by NDVI within a 250-m buffer, and cause-specific mortality: Compared with women in the lowest NDVI quintile, those in the highest quintile had 34% lower respiratory mortality (95% CI: 16–48%) and 13% lower cancer mortality (95% CI: 3–22%). The corresponding reductions were weaker in the second quintile, at 16% (95% CI: −4 to 31%) and 7% (95% CI: −3 to 16%), respectively (33). From a public health perspective, these findings suggest that anemia-related benefits may depend on reaching sufficient residential greenness exposure, rather than on small incremental increases at very low greenness levels.

This study found stronger inverse associations between residential greenness and anemia risk among women with normal BMI, those who were not breastfeeding, urban residents, and women with higher educational attainment. Several mechanisms may underlie this population heterogeneity. The weaker association among women with abnormal BMI may partly reflect differences in access to, use of, and physiological response to green spaces (34, 35). Women with normal BMI may be more likely to engage in outdoor physical activity and may have a more favorable metabolic and inflammatory profile, thereby enhancing the potential benefits of greenness (36). The attenuated association among breastfeeding women may reflect higher nutritional demands during lactation, which could weaken the observed association between residential greenness and anemia risk, as well as reduced time available for outdoor activity (37). The stronger association in urban areas may be related to better access to managed green spaces, healthcare resources, and health information, which could amplify the health benefits of greenness (38). Similarly, women with higher educational attainment may have greater health literacy and be more likely to use green spaces for physical activity and stress reduction, contributing to lower anemia risk.

Notably, we observed a U-shaped association between greenness and anemia by wealth index, with the most pronounced effect occurring in middle-income populations. Neighborhoods with higher socioeconomic status often have greater greenspace availability, including abundant vegetation coverage and better-planned green infrastructure (39). However, increased availability does not necessarily translate to greater exposure or use. Women from wealthier households may have less exposure to urban greenness because of busy schedules, preference for indoor recreational facilities, and reliance on air conditioning and private vehicles, which could attenuate the potential health benefits of greenness (40, 41). Conversely, women from poorer households may face challenges related to lower-quality green spaces, safety concerns, limited healthcare access, or higher background nutritional and infectious burdens, which may reduce the measurable benefits of greenness. These findings suggest that the health effects of residential greenness depend not only on the amount of surrounding vegetation, but also on socioeconomic context, accessibility, use patterns, and baseline vulnerability.

### 4.4 Implications for anemia prevention and public health strategies in LMICs

In our analysis, a substantial number of anemia cases were associated with low residential greenness under the hypothetical greenness improvement scenario. Consistent with previous studies (42, 43), we found that the spatial distributions of ANs and AFs were not fully aligned, reflecting the combined influence of exposure distribution, population density, and baseline disease prevalence. For instance, India had a relatively modest AF of 1.67%, but this corresponded to more than 1 million ANs because of its large population size and high anemia burden. In contrast, countries with smaller populations but limited greenness exposure, such as Peru and Jordan, had relatively high AFs of 4.62 and 4.19%, respectively, although their corresponding ANs were modest. These findings suggest that the anemia burden potentially associated with low residential greenness may vary substantially across LMIC settings with different demographic, epidemiological, and environmental characteristics. In densely populated regions with high baseline anemia prevalence, even modest improvements in residential greenness exposure may translate into considerable population-level benefits. In contrast, in settings with relatively limited greenness resources, improving the accessibility, quality and utilization of existing green spaces may represent a more feasible approach. These results highlight the importance of considering local population structure, disease burden, and environmental constraints when developing greenness-related public health strategies.

The choice of buffer size may also influence findings in greenness-related analyses. In Iran, the associations between greenness indices and risk of low birth weight were slightly attenuated when the exposure buffer was narrowed from 3,000 m to 500 m (6). A large-scale cohort study in China found that for each tertile increment in NDVI within 300-m, 500-m, and 1,000-m buffers, the risk of cardiovascular disease decreased by 16, 14, and 10%, respectively (44). The 1,000-m buffer is conceptually relevant to the “15-min city” planning model, which emphasizes access to essential public services and green spaces within a short walking or cycling distance (20). Supporting this scale, a time-series analysis in Hong Kong found that population-weighted NDVI significantly modified heat-mortality risks at buffers of 800–1,400 m, with the strongest association observed at 1000 m (25). However, findings from some studies suggest that buffer size may not significantly affect greenness accessibility (19, 31), possibly because of more uniform vegetation distribution, compact urban form, or limited scale sensitivity in specific settings. Therefore, buffer size should be interpreted not only as a technical modelling choice, but also as a proxy for the spatial scale at which residents may realistically interact with surrounding greenness.

Anemia remains a major global public health issue affecting women’s health and well-being. In response, WHO and the United Nations International Children’s Emergency Fund have set a target to halve the prevalence of anemia among WRA by 2030 (45). Our findings provide additional evidence that residential greenness may be relevant to anemia prevention strategies, particularly in LMICs where anemia burden remains high and environmental resources are unevenly distributed. To enhance the potential health benefits of urban greening, policymakers should consider not only increasing greenness coverage in residential environments, but also improving the quality, connectivity, safety, and accessibility of green spaces (46–48). Particular attention may be warranted in underserved and densely populated neighborhoods where green infrastructure is limited. Community-based strategies, such as public awareness initiatives and attractive social environments, can enhance greenness utilization for activities like physical exercise, thereby strengthening health benefits. Nevertheless, the feasibility of substantial greenness improvement may vary across regions, particularly in arid or climate-constrained settings where water availability and natural vegetation conditions may limit large-scale greening. Therefore, region-specific environmental and climatic conditions should also be considered when interpreting hypothetical greenness improvement scenarios and translating them into public health practice.

### 4.5 Strengths and limitations

Our study has several strengths. To our knowledge, this is the first multi-country analysis to systematically assess the association between residential greenness and anemia risk among WRA. By involving over one million participants, the study provides broad evidence across diverse demographic, socioeconomic, and environmental settings in LMICs. Greenness was measured using satellite-derived NDVI and EVI across various buffer sizes, enabling detailed analysis of spatial exposure patterns. In addition, we applied a counterfactual modelling framework to estimate the anemia burden potentially associated with low residential greenness, providing evidence to support targeted public health strategies.

There are several limitations in this study. The cross-sectional design limited our ability to infer causal relationships between residential greenness exposure and anemia risk. Therefore, the scenario-based burden estimates should be interpreted as hypothetical counterfactuals based on observed exposure–response associations. In addition, residential greenness may also be correlated with healthier food environments in some settings. Areas with greater greenness may have better access to fresh foods, community gardens, or local food markets, which could influence dietary quality and micronutrient intake relevant to anemia risk. Although detailed dietary information was unavailable in the present study, this pathway warrants further investigation in future studies. Finally, although DHS data provide standardized information across participating countries, differences in socioeconomic and healthcare conditions may limit the generalizability of our findings to regions not included in the analysis.

## 5 Conclusion

In conclusion, this large multi-country study found that higher residential greenness was associated with lower anemia risk among WRA across 40 LMICs. The association varied across socioeconomic and contextual subgroups. Hypothetical scenario-based analyses further suggested substantial cross-country variation in anemia burden potentially associated with low greenness exposure. These findings support residential greenness as a potentially relevant environmental determinant of anemia and highlight the need to consider local demographic, socioeconomic, and environmental contexts when integrating greenness into anemia prevention and environmental health strategies in LMICs.

## Data Availability

Publicly available datasets were analyzed in this study. This data can be found: Survey data used in this study are publicly available upon request from the DHS Program (https://dhsprogram.com). Greenness exposure was assessed using satellite-derived vegetation indices (NDVI and EVI) from the MODIS Vegetation Index Products (https://modis.gsfc.nasa.gov/data/dataprod/mod13.php). Gridded population data for WRA were obtained from the WorldPop age- and sex-structured datasets (https://hub.worldpop.org/geodata/listing?id=38). Country-specific prevalence of anemia among WRA was retrieved from the WHO Global Health Observatory database (https://data.who.int./zh/indicators/i/41D099F/8D58801).

## Footnotes

1
https://dhsprogram.com/
