# Supplementary material for: Association between residential greenness and anemia risk among women of reproductive age in low- and middle-income countries
Source: Front Public Health. 2026 Jul 6;14:1828024. doi: 10.3389/fpubh.2026.1828024 (PMC13383932; doi:10.3389/fpubh.2026.1828024)
Supplement: Supplementary file 1 [file Data_Sheet_1.docx]

Supplementary Material

**Table of Contents**

1. Text S1. List of included countries.

2. Text S2. Adjustment of hemoglobin.

3. Table S1. Distribution of greenness and PM_2.5_ among participants.

4. Table S2. Analysis of unadjusted and partially adjusted models.

5. Table S3. AF and AN of anemia cases in 40 countries in 2015.

6. Figure S1. Exclusion flowchart of the study population.

7. Figure S2. Spatial distributions of NDVI_-1000m_ and EVI_-1000m_.

8. Figure S3. Nonlinear exposure–response curves for greenness and anemia.

9. Figure S4. Stratified analysis of NDVI_-500m_ and EVI_-500m_.

10. Figure S5. Stratified analysis of NDVI_-2000m_ and EVI_-2000m_.

11. Figure S6. Stratified analysis of NDVI_-3000m_ and EVI_-3000m_.

12. Figure S7. Attributable fraction and number by achieving greenness target of anemia in 2015.

**Text S1**

The countries included are as follows: Albania, Armenia, Benin, Burkina Faso, Burundi, Cameroon, Côte d'Ivoire, Democratic Republic of the Congo, Eswatini, Ethiopia, Gabon, Gambia, Ghana, Guatemala, Guinea, Guyana, Haiti, Honduras, India, Jordan, Kyrgyzstan, Lesotho, Liberia, Madagascar, Malawi, Mali, Mozambique, Myanmar, Namibia, Niger, Nigeria, Peru, Rwanda, Senegal, Sierra Leone, South Africa, Togo, Uganda, United Republic of Tanzania, and Zimbabwe.

**Text S2**

According to the Guide to DHS Statistics, for altitudes below 1,000 meters, no adjustment is made. For altitudes above 1,000 meters, adjustment is made as follows:

$$\text{adj = -0.032*alt + 0.022*}\text{alt}^{\text{2}}$$

$$\text{adjHb}\text{ = Hb - adj if adj >0}$$

where adj stands for the amount to be adjusted, alt is the altitude in 1,000 feet, adjHb is the hemoglobin level adjusted for altitude, and Hb represents the measured value of hemoglobin in grams per deciliter.

**Table S1**

Distribution of greenness and PM_2.5_ among participants.

| **Exposures** | **Mean** | **SD** | **25^th^** | **Median** | **75^th^** | **Max** |
| --- | --- | --- | --- | --- | --- | --- |
| **All participants (n=1 074 790)** | | | | | | |
| PM_2.5_, (μg/m^3^) | 40.236 | 19.868 | 24.400 | 36.100 | 52.100 | 109.700 |
| NDVI_-500m_ | 0.461 | 0.141 | 0.377 | 0.461 | 0.548 | 0.875 |
| NDVI_-1000m_ | 0.463 | 0.137 | 0.382 | 0.461 | 0.546 | 0.872 |
| NDVI_-2000m_ | 0.465 | 0.133 | 0.387 | 0.462 | 0.546 | 0.865 |
| NDVI_-3000m_ | 0.468 | 0.130 | 0.392 | 0.464 | 0.548 | 0.851 |
| EVI_-500m_ | 0.287 | 0.091 | 0.232 | 0.288 | 0.340 | 0.692 |
| EVI_-1000m_ | 0.288 | 0.088 | 0.234 | 0.288 | 0.339 | 0.640 |
| EVI_-2000m_ | 0.290 | 0.085 | 0.238 | 0.288 | 0.339 | 0.610 |
| EVI_-3000m_ | 0.291 | 0.083 | 0.241 | 0.290 | 0.339 | 0.597 |
| **Incident anemia Cases (n=502 312)** | | | | | | |
| PM_2.5_, (μg/m^3^) | 42.824 | 20.302 | 26.800 | 39.500 | 57.600 | 109.700 |
| NDVI_-500m_ | 0.455 | 0.133 | 0.379 | 0.457 | 0.532 | 0.875 |
| NDVI_-1000m_ | 0.455 | 0.129 | 0.382 | 0.457 | 0.529 | 0.872 |
| NDVI_-2000m_ | 0.458 | 0.124 | 0.387 | 0.457 | 0.529 | 0.865 |
| NDVI_-3000m_ | 0.460 | 0.122 | 0.392 | 0.459 | 0.530 | 0.851 |
| EVI_-500m_ | 0.284 | 0.086 | 0.233 | 0.286 | 0.334 | 0.692 |
| EVI_-1000m_ | 0.284 | 0.083 | 0.236 | 0.286 | 0.332 | 0.640 |
| EVI_-2000m_ | 0.286 | 0.080 | 0.239 | 0.286 | 0.331 | 0.610 |
| EVI_-3000m_ | 0.287 | 0.078 | 0.242 | 0.287 | 0.331 | 0.597 |
| **Non-cases (n=572 478)** | | | | | | |
| PM_2.5_, (μg/m^3^) | 37.964 | 19.195 | 23.200 | 33.500 | 47.200 | 109.700 |
| NDVI_-500m_ | 0.468 | 0.147 | 0.376 | 0.465 | 0.564 | 0.875 |
| NDVI_-1000m_ | 0.469 | 0.144 | 0.381 | 0.465 | 0.563 | 0.872 |
| NDVI_-2000m_ | 0.472 | 0.139 | 0.387 | 0.467 | 0.563 | 0.865 |
| NDVI_-3000m_ | 0.476 | 0.137 | 0.392 | 0.469 | 0.566 | 0.851 |
| EVI_-500m_ | 0.290 | 0.095 | 0.230 | 0.289 | 0.347 | 0.692 |
| EVI_-1000m_ | 0.291 | 0.092 | 0.233 | 0.290 | 0.346 | 0.640 |
| EVI_-2000m_ | 0.293 | 0.089 | 0.237 | 0.291 | 0.346 | 0.610 |
| EVI_-3000m_ | 0.295 | 0.087 | 0.240 | 0.292 | 0.347 | 0.597 |

SD Standard deviation, PM_2.5_ particulate matter with a diameter of ≤2.5 μm, NDVI normalized difference vegetation index, EVI enhanced vegetation index.

**Table S2**

Analysis of unadjusted and partially adjusted models.

|  | **Model1** | |  | **Model2** | |
| --- | --- | --- | --- | --- | --- |
|  | **OR (95% CI)** | **P** |  | **OR (95% CI)** | **P** |
| **NDVI_-500_** |  |  |  |  |  |
| Tertile 1 | Ref. (1) | **-** |  | Ref. (1) | **-** |
| Tertile 2 | 1.008 (0.998, 1.019) | 0.121 |  | 0.994 (0.984, 1.004) | 0.253 |
| Tertile 3 | 0.825 (0.816, 0.834) | <0.001 |  | 0.873 (0.863, 0.883) | <0.001 |
| Per 0.1-unit increment | 0.952 (0.949, 0.955) | <0.001 |  | 0.937 (0.934, 0.940) | <0.001 |
| **NDVI_-1000m_** |  |  |  |  |  |
| Tertile 1 | Ref. (1) | - |  | Ref. (1) | - |
| Tertile 2 | 1.005 (0.995, 1.016) | 0.302 |  | 0.994 (0.984, 1.005) | 0.284 |
| Tertile 3 | 0.819 (0.81, 0.828) | <0.001 |  | 0.868 (0.858, 0.878) | <0.001 |
| Per 0.1-unit increment | 0.947 (0.944, 0.95) | <0.001 |  | 0.932 (0.929, 0.935) | <0.001 |
| **NDVI_-2000_** |  |  |  |  |  |
| Tertile 1 | Ref. (1) | **-** |  | Ref. (1) | **-** |
| Tertile 2 | 1.004 (0.994, 1.014) | 0.429 |  | 0.994 (0.984, 1.004) | 0.263 |
| Tertile 3 | 0.809 (0.801, 0.818) | <0.001 |  | 0.859 (0.849, 0.869) | <0.001 |
| Per 0.1-unit increment | 0.938 (0.934, 0.941) | <0.001 |  | 0.923 (0.920, 0.927) | <0.001 |
| **NDVI_-3000_** |  |  |  |  |  |
| Tertile 1 | Ref. (1) | **-** |  | Ref. (1) | **-** |
| Tertile 2 | 1.001 (0.991, 1.012) | 0.780 |  | 0.994 (0.983, 1.004) | 0.216 |
| Tertile 3 | 0.795 (0.786, 0.804) | <0.001 |  | 0.844 (0.835, 0.854) | <0.001 |
| Per 0.1-unit increment | 0.929 (0.926, 0.932) | <0.001 |  | 0.917 (0.913, 0.920) | <0.001 |
| **EVI_-500_** |  |  |  |  |  |
| Tertile 1 | Ref. (1) | **-** |  | Ref. (1) | **-** |
| Tertile 2 | 1.008 (0.998, 1.018) | 0.131 |  | 0.99 (0.979, 1) | 0.049 |
| Tertile 3 | 0.866 (0.856, 0.875) | <0.001 |  | 0.89 (0.88, 0.9) | <0.001 |
| Per 0.1-unit increment | 0.949 (0.944, 0.954) | <0.001 |  | 0.924 (0.920, 0.929) | <0.001 |
| **EVI_-1000m_** |  |  |  |  |  |
| Tertile 1 | Ref. (1) | - |  | Ref. (1) | - |
| Tertile 2 | 0.99 (0.98, 1) | 0.048 |  | 0.971 (0.961, 0.981) | <0.001 |
| Tertile 3 | 0.851 (0.842, 0.86) | <0.001 |  | 0.877 (0.867, 0.886) | <0.001 |
| Per0.1-unit increment | 0.941 (0.937, 0.946) | <0.001 |  | 0.916 (0.911, 0.921) | <0.001 |
| **EVI_-2000_** |  |  |  |  |  |
| Tertile 1 | Ref. (1) | **-** |  | Ref. (1) | **-** |
| Tertile 2 | 0.997 (0.987, 1.007) | 0.549 |  | 0.978 (0.968, 0.988) | <0.001 |
| Tertile 3 | 0.844 (0.835, 0.853) | <0.001 |  | 0.872 (0.862, 0.881) | <0.001 |
| Per 0.1-unit increment | 0.928 (0.923, 0.933) | <0.001 |  | 0.904 (0.899, 0.908) | <0.001 |
| **EVI_-3000_** |  |  |  |  |  |
| Tertile 1 | Ref. (1) | **-** |  | Ref. (1) | **-** |
| Tertile 2 | 0.996 (0.986, 1.007) | 0.493 |  | 0.976 (0.966, 0.986) | <0.001 |
| Tertile 3 | 0.834 (0.825, 0.844) | <0.001 |  | 0.864 (0.854, 0.873) | <0.001 |
| Per 0.1-unit increment | 0.915 (0.910, 0.920) | <0.001 |  | 0.894 (0.889, 0.898) | <0.001 |

OR Odds ratio, CI Confidence interval, NDVI normalized difference vegetation index, EVI enhanced vegetation index

Model 1: a crude model without covariate adjustment.

Model 2: adjusted for age, BMI (body mass index), highest education level, marital status, pregnant, breastfeeding.

**Table S3**

Attributable fraction (95%CI) and number (95%CI) by achieving greenness targets of mean NDVI/EVI estimates of anemia across 40 countries in 2015.

| **Country** | **NDVI_-1000m_** | |  | **EVI_-1000m_** | |
| --- | --- | --- | --- | --- | --- |
|  | **AF (95%CI)** | **AN (95%CI)** |  | **AF (95%CI)** | **AN (95%CI)** |
| Albania | 3.01% (1.47%, 4.53%) | 1554 (760, 2340) |  | 2.55% (0.89%, 4.20%) | 1321 (460, 2178) |
| Armenia | 4.03% (2.48%, 5.56%) | 4289 (2644, 5924) |  | 3.52% (1.95%, 5.08%) | 3758 (2082, 5432) |
| Benin | 1.63% (0.78%, 2.46%) | 7688 (3694, 11636) |  | 1.58% (0.65%, 2.51%) | 6004 (2460, 9522) |
| Burkina Faso | 2.30% (1.45%, 3.14%) | 50591 (32008, 69053) |  | 1.76% (0.82%, 2.71%) | 36942 (17098, 56732) |
| Burundi | 2.30% (0.96%, 3.63%) | 933 (390, 1470) |  | 2.16% (0.72%, 3.60%) | 996 (331, 1659) |
| Cameroon | 2.36% (1.22%, 3.50%) | 25428 (13087, 37640) |  | 1.89% (0.60%, 3.17%) | 16554 (5257, 27789) |
| DR Congo | 2.90% (1.95%, 3.85%) | 4397 (2953, 5835) |  | 2.69% (1.73%, 3.65%) | 4114 (2651, 5578) |
| Cote d'lvoire | 2.66% (1.94%, 3.36%) | 24829 (18182, 31455) |  | 2.59% (1.85%, 3.33%) | 22240 (15894, 28579) |
| Ethiopia | 3.10% (1.63%, 4.56%) | 61293 (32166, 90123) |  | 2.77% (1.17%, 4.37%) | 60272 (25398, 95009) |
| Gabon | 1.58% (0.69%, 2.46%) | 1189 (518, 1853) |  | 1.38% (0.35%, 2.40%) | 1066 (270, 1857) |
| Gambia | 1.75% (0.86%, 2.63%) | 2768 (1360, 4162) |  | 1.55% (0.57%, 2.53%) | 2027 (742, 3304) |
| Ghana | 2.64% (1.54%, 3.73%) | 34485 (20070, 48782) |  | 2.27% (1.09%, 3.45%) | 26637 (12759, 40457) |
| Guatemala | 3.59% (1.97%, 5.20%) | 1373 (754, 1986) |  | 3.17% (1.26%, 5.07%) | 1279 (508, 2046) |
| Guinea | 2.26% (1.34%, 3.19%) | 7049 (4156, 9921) |  | 2.19% (1.27%, 3.11%) | 7333 (4241, 10417) |
| Guyana | 2.53% (1.23%, 3.82%) | 329 (160, 497) |  | 2.30% (0.88%, 3.72%) | 204 (78, 330) |
| Haiti | 2.39% (1.40%, 3.37%) | 11273 (6621, 15889) |  | 2.02% (0.97%, 3.06%) | 7988 (3834, 12128) |
| Honduras | 3.20% (1.53%, 4.86%) | 1123 (536, 1703) |  | 2.60% (0.77%, 4.42%) | 846 (250, 1440) |
| India | 1.67% (0.76%, 2.57%) | 1017063 (461298, 566167) |  | 1.52% (0.49%, 2.54%) | 892763 (287804, 1494247) |
| Jordan | 4.19% (3.31%, 5.07%) | 22320 (17631, 27015) |  | 3.81% (2.85%, 4.78%) | 20184 (15072, 25305) |
| Kyrgyzstan | 3.20% (2.04%, 4.36%) | 15404 (9809, 20967) |  | 2.93% (1.75%, 4.10%) | 13446 (8049, 18829) |
| Lesotho | 2.46% (0.99%, 3.92%) | 3169 (1271, 5040) |  | 2.50% (0.96%, 4.04%) | 3545 (1365, 5724) |
| Liberia | 1.94% (0.80%, 3.07%) | 1969 (809, 3113) |  | 2.08% (0.90%, 3.25%) | 2157 (934, 3375) |
| Madagascar | 1.65% (0.38%, 2.89%) | 10674 (2473, 18750) |  | 1.58% (0.11%, 3.05%) | 14833 (989, 28574) |
| Malawi | 1.53% (0.21%, 2.83%) | 4792 (644, 8876) |  | 1.44% (-0.29%, 3.14%) | 6167 (-1257, 13507) |
| Mali | 2.14% (1.42%, 2.85%) | 44554 (29589, 59458) |  | 1.79% (1.01%, 2.57%) | 34964 (19762, 50133) |
| Mozambique | 1.58% (0.53%, 2.61%) | 9070 (3040, 15015) |  | 1.40% (0.20%, 2.58%) | 9255 (1342, 17111) |
| Myanmar | 1.74% (0.55%, 2.91%) | 27670 (8748, 46315) |  | 1.56% (0.17%, 2.94%) | 22111 (2432, 41624) |
| Namibia | 4.42% (3.25%, 5.58%) | 6499 (4782, 8217) |  | 3.56% (2.24%, 4.87%) | 5105 (3213, 6998) |
| Niger | 3.31% (2.62%, 4.00%) | 67136 (53217, 81133) |  | 2.73% (1.93%, 3.52%) | 55204 (39113, 71337) |
| Nigeria | 2.04% (1.21%, 2.87%) | 289745 (171701, 406878) |  | 1.61% (0.68%, 2.54%) | 197874 (83506, 311806) |
| Peru | 4.62% (3.66%, 5.57%) | 59171 (46891, 71421) |  | 5.05% (4.09%, 6.01%) | 65232 (52807, 77650) |
| Rwanda | 2.27% (0.50%, 4.01%) | 556 (123, 982) |  | 2.13% (0.20%, 4.04%) | 597 (56, 1134) |
| Senegal | 2.51% (1.76%, 3.26%) | 40760 (28604, 52874) |  | 1.93% (1.08%, 2.78%) | 30157 (16885, 43399) |
| Sierra Leone | 2.32% (1.35%, 3.27%) | 3614 (2103, 5111) |  | 2.29% (1.34%, 3.24%) | 3687 (2157, 5213) |
| South Africa | 3.14% (1.78%, 4.50%) | 92573 (52467, 132347) |  | 2.82% (1.41%, 4.22%) | 89497 (44892, 134037) |
| Eswatini | 1.40% (0.08%, 2.70%) | 135 (8, 261) |  | 1.39% (-0.37%, 3.13%) | 308 (-82, 694) |
| Tanzania | 2.20% (1.06%, 3.33%) | 30108 (14501, 45544) |  | 1.89% (0.57%, 3.20%) | 26397 (7927, 44748) |
| Togo | 1.94% (0.90%, 2.95%) | 7563 (3534, 11545) |  | 1.70% (0.54%, 2.86%) | 5924 (1888, 9936) |
| Uganda | 2.03% (0.63%, 3.41%) | 3275 (1013, 5503) |  | 2.00% (0.45%, 3.54%) | 3923 (888, 6941) |
| Zimbabwe | 1.85% (0.40%, 3.28%) | 6733 (1449, 11932) |  | 1.42% (-0.36%, 3.17%) | 6111 (-1549, 13692) |

NDVI normalized difference vegetation index, EVI enhanced vegetation index, AF Attributable fraction, AN Attributable number, CI Confidence interval.

Model adjusted for age, body mass index, highest education level, marital status, pregnant, breastfeeding, residence, wealth index, family size, age of household head, gender of household head, health insurance covered, type of cooking energy, type of drinking water, type of toilet and PM_2.5_.


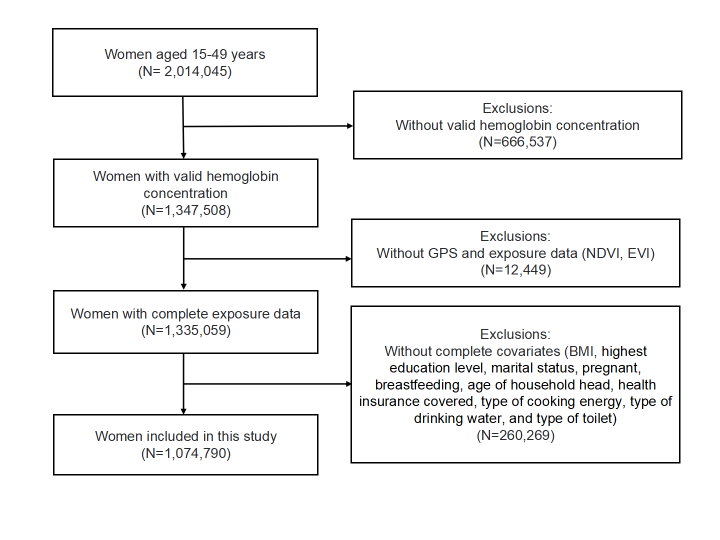


**Figure S1**

Exclusion flowchart of population included in this study. NDVI normalized difference vegetation index, EVI enhanced vegetation index, BMI body mass index.

**
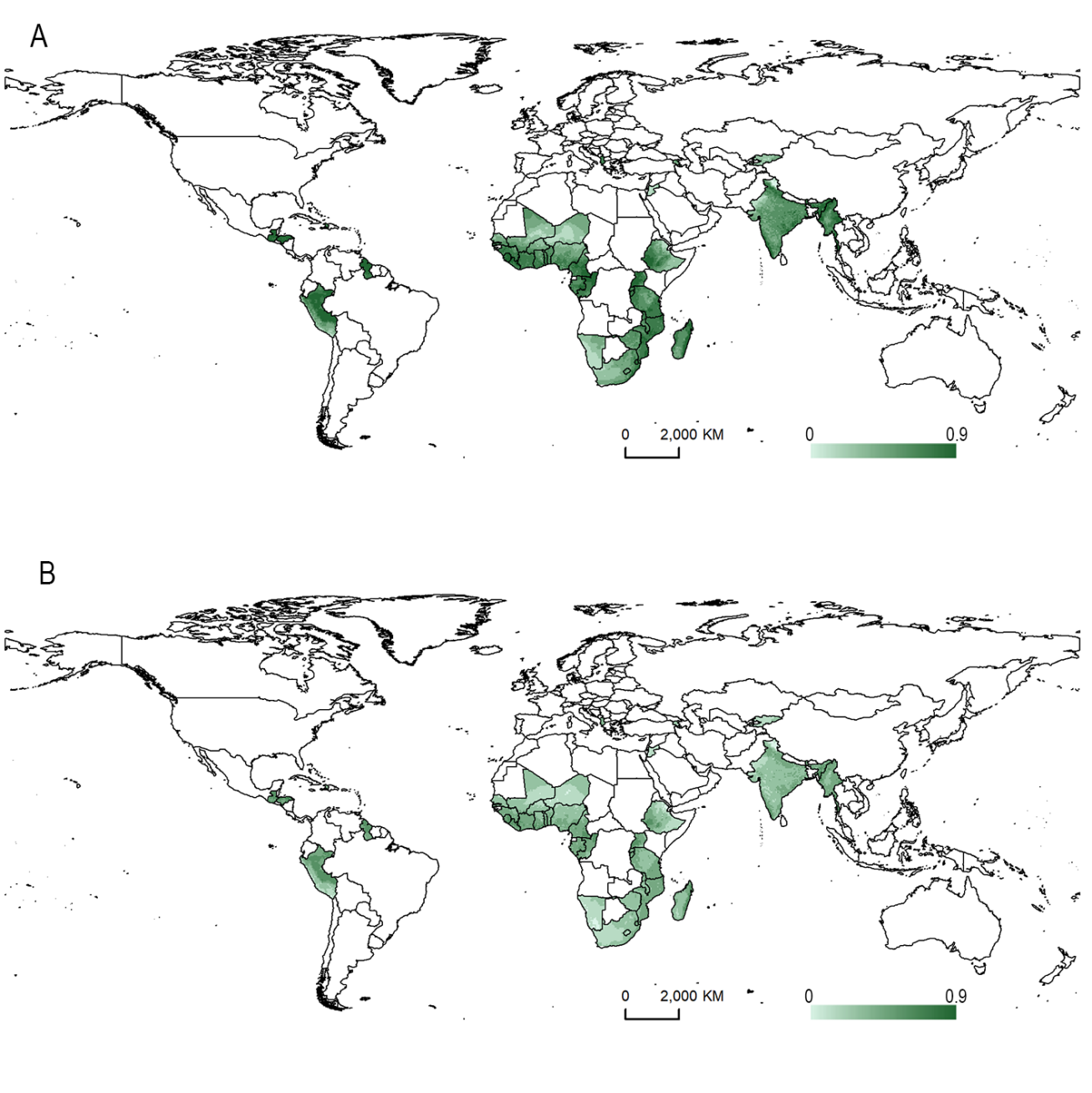
**

**Figure S2**

The spatial distributions of NDVI_-1000m_ (A) and EVI_-1000m_ (B) for countries surveyed in this study. NDVI normalized difference vegetation index, EVI enhanced vegetation index.


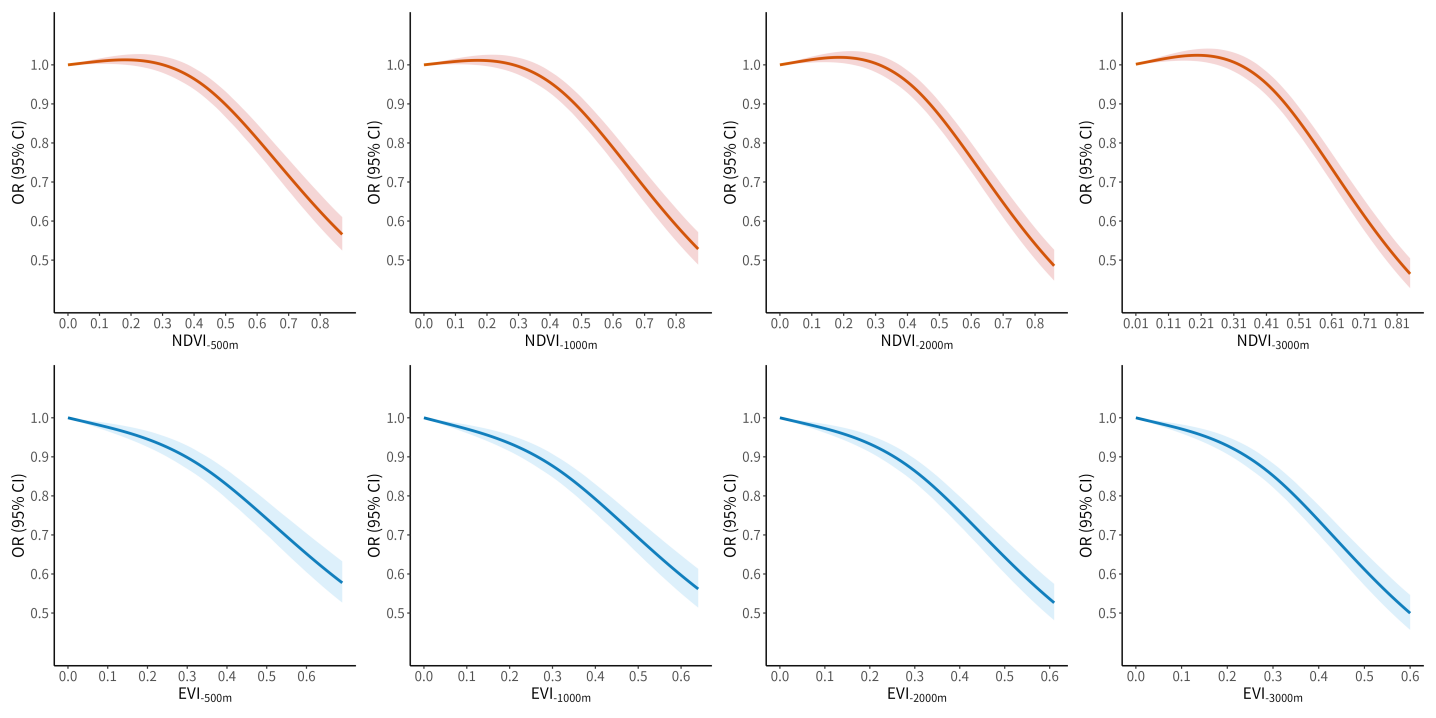


**Figure S3**

Nonlinear exposure-response function (Nonlinear P<0.001) of greenness exposure (NDVI_-500m_, NDVI_-1000m,_ NDVI_-2000m_, NDVI_-3000m_, EVI_-500m_, EVI_-1000m,_ EVI_-2000m_ and EVI_-3000m_) and anemia. NDVI normalized difference vegetation index, EVI enhanced vegetation index, OR Odds ratio, CI Confidence interval.


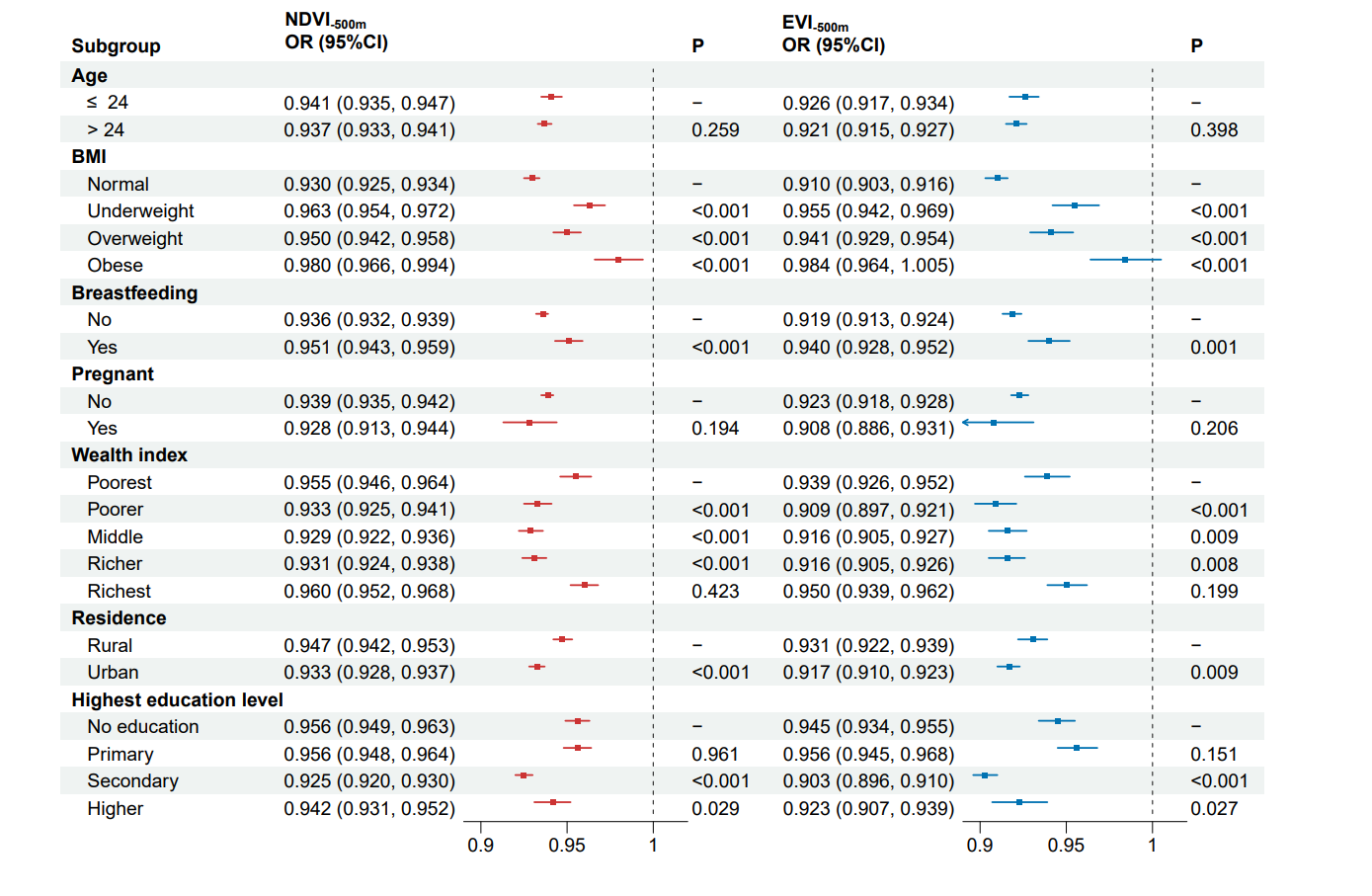


**Figure S4**

Stratified analysis of NDVI_-500m_ and EVI_-500m._ NDVI normalized difference vegetation index, EVI enhanced vegetation index, OR Odds ratio, CI Confidence interval.


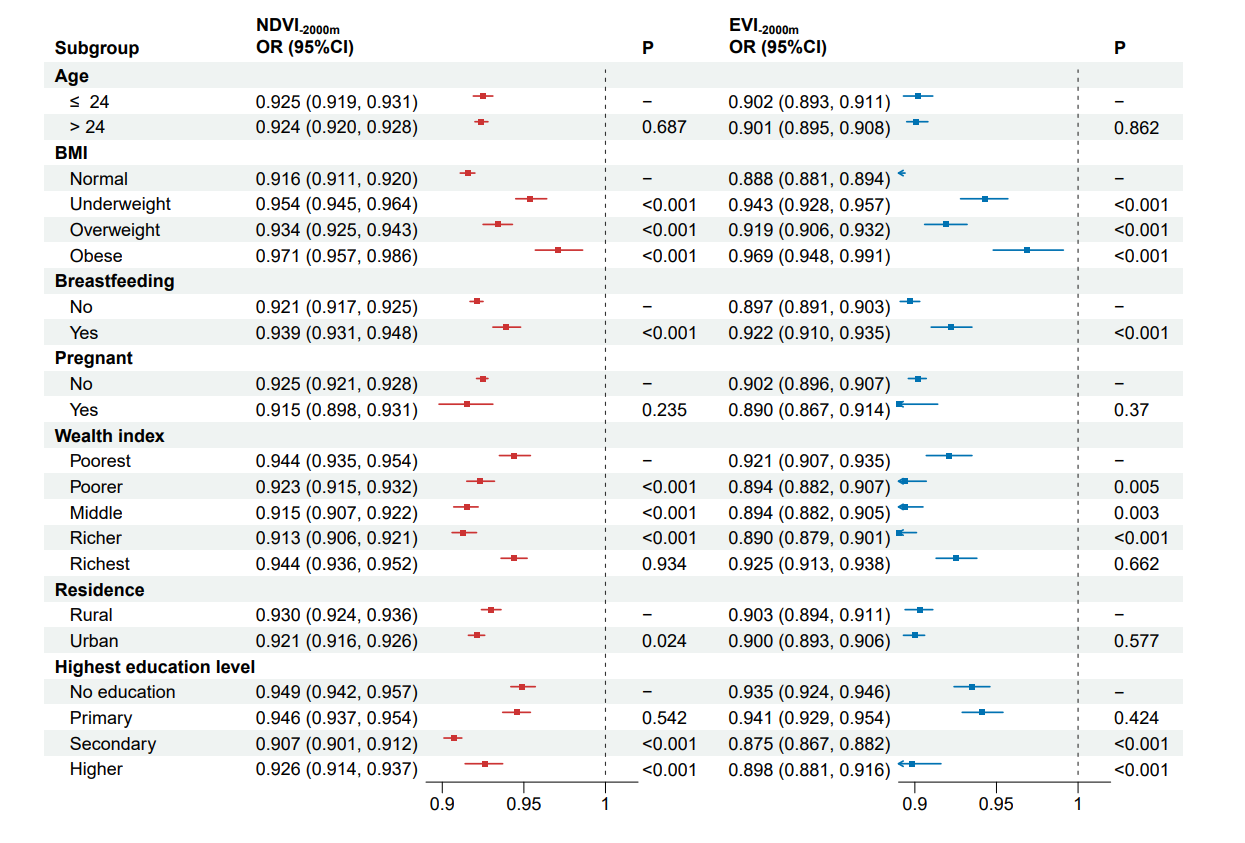


**Figure S5**

Stratified analysis of NDVI_-2000m_ and EVI_-2000m._ NDVI normalized difference vegetation index, EVI enhanced vegetation index, OR Odds ratio, CI Confidence interval.


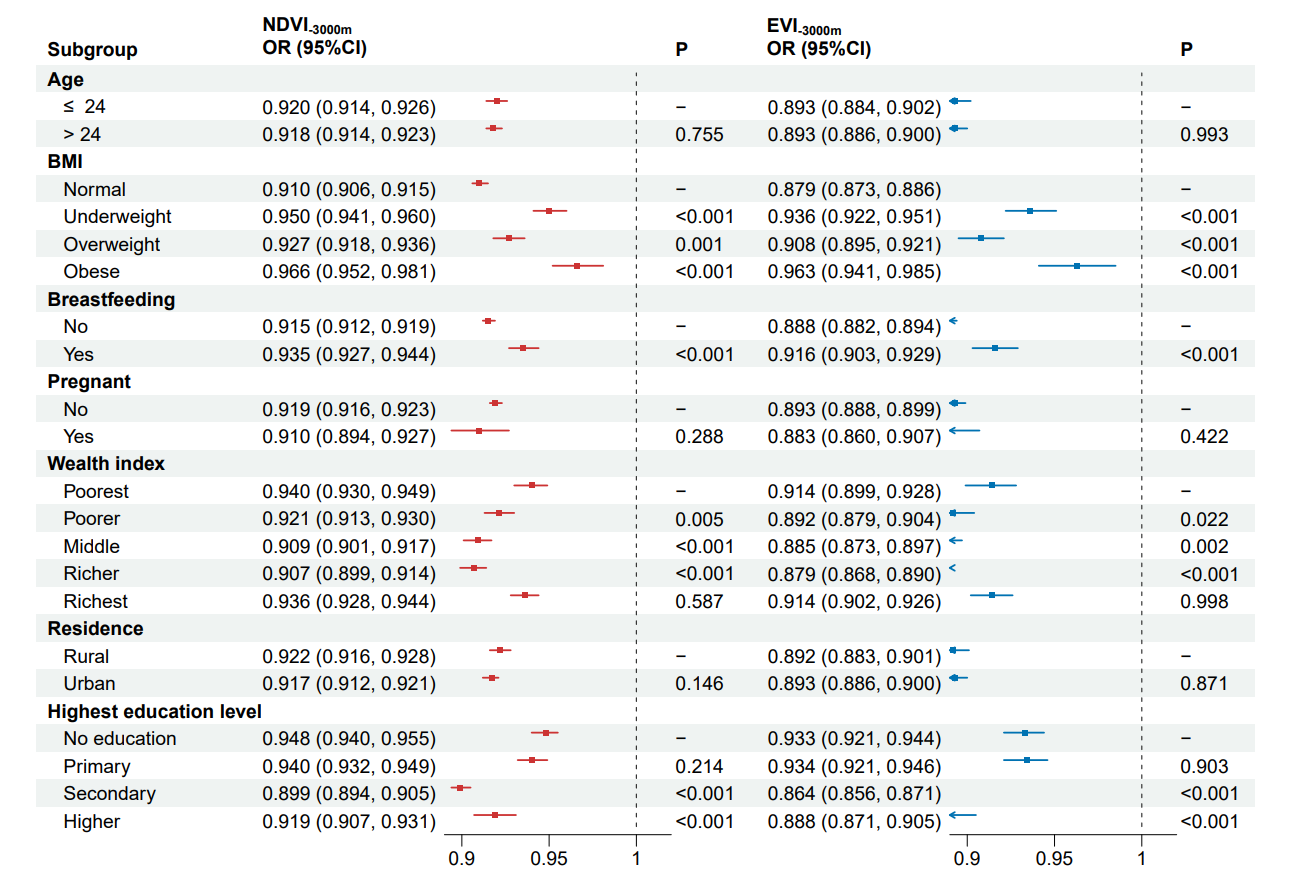


**Figure S6**

Stratified analysis of NDVI_-3000m_ and EVI_-3000m._ NDVI normalized difference vegetation index, EVI enhanced vegetation index, OR Odds ratio, CI Confidence interval.


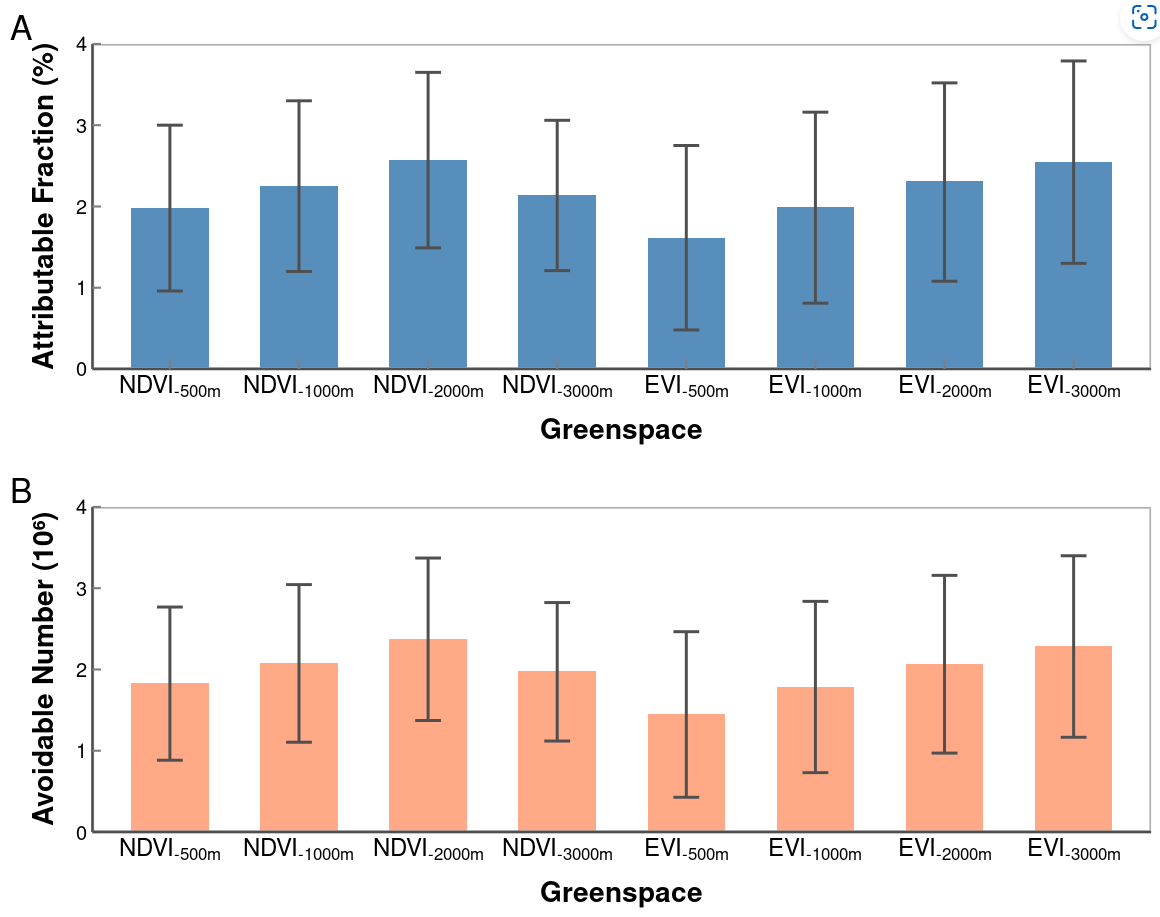


**Figure S7**

Overall burden estimates of anemia in 40 low- and middle-income countries in 2015. Attributable fraction (A) and number (B) by achieving greenness target of anemia. Error bars represent 95% confidence intervals. NDVI normalized difference vegetation index, EVI enhanced vegetation index.
